# Statin non-adherence: clinical consequences and proposed solutions

**DOI:** 10.12688/f1000research.8215.1

**Published:** 2016-04-21

**Authors:** Robert S. Rosenson

**Affiliations:** 1 Department of Medicine, Icahn School of Medicine at Mount Sinai and Mount Sinai Heart, New York, NY, USA

**Keywords:** statin therapy, cardiovascular events, coronary heart disease, acute coronary syndromes, statin therapy adherence

## Abstract

Large controlled clinical trials have demonstrated reductions with statin therapy in cardiovascular events in patients presenting with acute coronary syndromes and stable coronary heart disease and individuals at high risk of a cardiovascular event. In trials of acute coronary syndromes and stable coronary heart disease, high-intensity statin therapy is more effective in the prevention of recurrent cardiovascular events than low-intensity statin therapy. Thus, evidence-based guidelines recommend in-hospital initiation of high-intensity statin therapy for all acute coronary syndrome patients. Clinical trials report high adherence to and low discontinuation of high-intensity statin therapy; however, in clinical practice, high-intensity statins are prescribed to far fewer patients, who often discontinue their statin after the first refill. A coordinated effort among the patient, provider, pharmacist, health system, and insurer is necessary to improve utilization and persistence of prescribed medications. The major cause for statin discontinuations reported by patients is perceived adverse events. Evaluation of potential adverse events requires validated tools to distinguish between statin-associated adverse events versus non-specific complaints. Treatment options for statin-intolerant patients include the use of a different statin, often at a lower dose or frequency. In order to lower LDL cholesterol, lower doses of statins may be combined with ezetimibe or bile acid sequestrants. Newer treatment options for patients with statin-associated muscle symptoms may include proprotein convertase subtilisin kexin 9 (PCSK9) inhibitors.

## Introduction

Among patients hospitalized for an acute coronary syndrome (ACS) and stable coronary heart disease (CHD), randomized controlled trials have demonstrated that high-dose/high-intensity atorvastatin therapy is more effective than placebo, low-intensity therapy with pravastatin, moderate-intensity therapy with simvastatin, or low-dose atorvastatin therapy in the reduction of recurrent cardiovascular disease (CVD) events ^1^. In a meta-analysis, high-intensity statin therapy was more effective than control or low-moderate intensity for reducing the risk of vascular death (1.3% versus 1.5%, [0.88 {0.84–0.91}]) and all-cause mortality (2.3% versus 2.5%, [0.91{0.88–0.93}]) ^2^. Thus, the American College of Cardiology/American Heart Association (ACC/AHA) guidelines for the treatment of ACS and secondary prevention of CHD recommend the initiation of high-intensity statin therapy in patients with clinical atherosclerotic CVD (ASCVD) regardless of baseline low-density lipoprotein (LDL) cholesterol levels ^2^. In contrast, European Society of Cardiology/European Atherosclerosis Society (ESC/EAS) guidelines recommend reductions in LDL cholesterol to less than 1.8 mmol/L or by 50% or more ^3^. The National Lipid Association (NLA) and International Atherosclerosis Society adopted a similar LDL cholesterol-centric perspective and recommend LDL cholesterol levels less than 1.8 mmol/L regardless of the statin dosage needed to achieve this target ^4, 5^. From the perspective of randomized clinical trials, the mandate for empiric high-intensity statins is particularly relevant for ACS patients in whom initiation of this therapy is recommended before hospital discharge ^6^.

Data from ACS registries suggest that over 80% of patients are prescribed statins following a myocardial infarction (MI) or coronary revascularization ^7^. However, few prior studies have reported the percentage of patients who filled prescriptions for high-intensity statins following CHD events. In ACS registries conducted from 2003 to 2008, utilization of statins ranged from 80 to 91%, while only 23–38% were prescribed high-intensity statins. In a real-world analysis of Medicare beneficiaries, which included hospitalizations for CHD between 2007 and 2011, only 27% with insurance coverage for medications were prescribed high-intensity statins after hospitalization for a coronary event ^8^. The principal factor associated with being discharged and remaining on high-intensity statin therapy for 365 days was prior use of a high-intensity statin. These data suggest that clinicians focus on the LDL cholesterol rather than the clinical trial evidence supporting high-intensity statins that encompasses other atherothrombotic properties ^9^. Utilization of high-intensity statins in Medicare beneficiaries diminished progressively during the ensuing year, such that an additional 24% of participants reduced their statin dosage or discontinued high-intensity statins ^8^. Among patients hospitalized for a non-cardiovascular illness, who then have an in-hospital myocardial infarction, the use of high-intensity statins is lower ^10^. At the time of this survey, simvastatin 80 mg was the only generic statin. Due to the safety concerns with this dosage of simvastatin, particularly in the elderly and in those patients taking multiple medications, simvastatin 40 mg daily was the more commonly prescribed dosage.

Utilization and persistence of high-intensity statins represents an important performance measure ^6^; however, the clinical consequences of non-adherence to high-intensity statin therapy have been less well studied. In preliminary data from a 5% sample Medicare population, poor adherence and discontinuation of high-intensity statin therapy after the first prescription fill was accompanied by higher rates of hospitalization for cardiovascular and non-cardiovascular causes and more deaths during the ensuing 5 years ^11^.

Since hospitalizations for recurrent cardiovascular events increase more rapidly in patients hospitalized for a MI than age- and sex-matched controls hospitalized for other causes, short-term and long-term secondary preventive measures are crucial to minimize the risk of recurrent events ^12^. Thus, discontinuation of statins and other evidence-based secondary preventive therapies has implications for the patient’s future health as well as economic costs to the patient, their family, and society.

Several reasons contribute to statin down-titration or discontinuation. In clinical trials, statin-associated adverse events (statin-associated muscle symptoms [SAMS]) are no different between participants assigned to statins or placebo ^13^. However, clinical trials select individuals with lower risk for muscle events based on age, prior musculoskeletal complaints, renal function, and concomitant non-drug and drug therapies that interact with drug elimination pathways. Discontinuation of statins is more common among patients with side effects, which were reported by 60% of former users and 25% of current users ^14^. Since statin intolerance is often symptom based, it is important to develop and administer validated measures of statin intolerance. The NLA proposed a clinical tool for the assessment of SAMS ^15^ that was based on a retrospective analysis of the STOMP trial, which investigated the effects of statin medication on muscle performance ^16^. In order to improve the accuracy of diagnosis of an adverse event, this index incorporates the fundamental process of de-challenge and re-challenge with either the same statin at a lower dosage or an alternate statin that has different drug elimination pathways that may be genetically based.

Non-statin LDL cholesterol-lowering therapies have been evaluated in patients who report SAMS. These studies have randomized individuals who experienced SAMS with two statins that included one agent at the lowest approved dosages. Second-line LDL cholesterol-lowering agents often used in patients who experience SAMS include ezetimibe, bile acid sequestrants, and niacin. However, these agents have modest LDL cholesterol-lowering efficacy. In trials with fully human monoclonal antibodies to proprotein convertase subtilisin kexin 9 (PCSK9) inhibitors (alirocumab and evolocumab), SAMS were reported in fewer individuals and no more often than when treated with ezetimibe ^17– 19^. The mean LDL cholesterol reduction was 53–56% with evolocumab compared with 15–18% with ezetimibe ^17, 18^. Alirocumab reduced LDL cholesterol by 45% compared to 15% with ezetimibe ^19^. Bempedoic acid (ETC-1002) inhibits ATP citrate lyase (ACL), a key enzyme that supplies substrate for cholesterol and fatty acid synthesis in the liver. Since bempedoic acid inhibits an enzyme earlier in the cholesterol synthetic pathway than statins, it is possible that adverse muscle symptoms caused by HMG-CoA reductase inhibition would also occur with drugs that inhibit metabolites such as mevalonate even earlier in the synthetic pathway. This agent was evaluated in a subgroup of 56 SAMS participants (37 ETC-1002 group and 19 placebo group) enrolled in a larger randomized, placebo-controlled trial; the mean difference in LDL cholesterol was 28.7% versus placebo ^20^. Adverse muscle complaints were similar in the placebo and ETC-1002 treatment groups.

These trials have not incorporated a placebo-controlled challenge and re-challenge phase with statin therapy to ensure appropriate identification of SAMS individuals. The ODYSSEY ALTERNATIVE included a single-blind placebo run-in and excluded participants who reported SAMS with placebo ^19^. In the second phase, continuing participants were randomized to double-blind treatment (2:2:1) with alirocumab, ezetimibe 10 mg daily, or atorvastatin 20 mg daily. On re-exposure to statin therapy, nearly 50% tolerated atorvastatin. SAMS were 39% less frequent with alirocumab versus atorvastatin. The GAUSS III trial design incorporated the fundamental concept for evaluation of adverse drug reactions through a randomized challenge and de-challenge treatment with placebo and atorvastatin 20 mg daily ^21^. Thus, this trial design may serve as a state-of-the-art model for future such trials in SAMS patients. GAUSS III enrolled 511 patients with uncontrolled LDL cholesterol and history of intolerance to two or more statins ^22^. 

Currently, the phase III clinical outcome trials with PCSK9 inhibitors have not been reported ^23^. The first large cardiovascular outcome trial with a PCSK9 inhibitor is expected to announce results in the second half of 2016 ^24^. These trials have enrolled patients with cardiovascular disease who have elevated LDL cholesterol levels on moderate- to high-intensity statins and therefore do not address clinical outcomes in patients treated with PCSK9 monotherapy.

## Conclusions

Non-adherence to evidence-based secondary preventive LDL cholesterol-lowering statin therapies increases the risk for recurrent cardiovascular and non-cardiovascular events, and all-cause mortality. Coordinated efforts to improve adherence involve patient factors, provider behavior, and health system factors. Many patients who discontinue their medications perceive that non-specific complaints are drug related and then decide to terminate treatment on their own initiative without the input of healthcare professionals. Thus, it is important to engage in a dialogue concerning major adverse reactions with any medication and evaluate reported side effects with an objective clinical tool, such as the statin muscle index. Non-statin approaches are second-line therapies to LDL cholesterol, but they are ineffective in either lowering LDL cholesterol levels by more than 50% or achieving LDL cholesterol levels that meet established targets proposed by several consensus documents. Anti-PCSK9 antibodies are more effective LDL cholesterol-lowering agents than ezetimibe in multiple short-term studies. The efficacy, safety, tolerability, and long-term persistence of PCSK9 inhibitors await completion of large ongoing clinical trials. These trials have enrolled patients with cardiovascular disease, including ACS, who have LDL cholesterol levels ≥70 mg/dL on maximally tolerated moderate- to high-intensity statins. These trials did not specifically include patients with SAMS. Future trials with PCSK9 inhibitor monotherapy will be required to address the unmet need in high-risk patients who refuse or cannot tolerate statin treatment.
